# Psychoneuroendocrine stress response in female and male youth with major depressive disorder

**DOI:** 10.1111/jcpp.14168

**Published:** 2025-03-23

**Authors:** Anka Bernhard, Nikola Fann, Andreas G. Chiocchetti, Katharina Ackermann, Anne Martinelli, Christine M. Freitag

**Affiliations:** ^1^ Department of Child and Adolescent Psychiatry, Psychosomatics and Psychotherapy University Hospital Frankfurt am Main, Goethe University Frankfurt am Main Germany; ^2^ Department of Child and Adolescent Psychiatry and Psychotherapy Faculty of Medicine, Technische Universität Dresden, German Center for Child and Adolescent Health (DZKJ), Partner Site Leipzig/Dresden Dresden Germany; ^3^ Faculty of Education, University Hamburg Hamburg Germany; ^4^ Fresenius University of Applied Sciences Frankfurt am Main Frankfurt am Main Germany

**Keywords:** Major depressive disorder, adolescence, stress response, cortisol, testosterone, oxytocin

## Abstract

**Background:**

Exposure to psychosocial stress is one of the strongest risk factors for major depressive disorder (MDD) in youth, but underlying neurobiological mechanisms are poorly understood. Previous studies on the neuroendocrine stress response in youth with MDD are scarce, limited to cortisol, and rarely considered sex differences. Due to puberty‐associated neuroendocrine transitions increasing the risk for MDD onset in adolescence, this study aimed to investigate sex‐specific stress responses of stress and sex hormones as well as of neuropeptides.

**Methods:**

In 103 pubertal youths with MDD and 72 healthy controls (HCs; 62% females, 12–18 years), psychological stress as well as salivary cortisol, testosterone, and oxytocin reactivity to a standardized psychosocial stress test (Trier Social Stress Test, TSST) were assessed. Effects of group and sex, and their interactions were analyzed using hierarchical linear models, while controlling for potentially confounding factors (such as age and pubertal status).

**Results:**

Females and males with MDD showed a stronger psychological stress response than HCs. In contrast, both female and male youth with MDD showed blunted cortisol, testosterone, and oxytocin stress responses compared to HCs. In addition, baseline testosterone was elevated in MDD compared to HCs.

**Conclusions:**

Results indicate a discrepant stress reactivity in youth with MDD, with increased psychological, but decreased neuroendocrine responses to psychosocial stress. Blunted neuroendocrine stress responses in youth with MDD were found across different neuroendocrine systems and in both females and males with MDD. These novel findings point to a fundamentally changed stress response in youth with MDD irrespective of sex, which may influence successful stress regulation in the affected adolescents.

## Introduction

Major depressive disorder (MDD) covers a variety of symptoms related to substantial changes in affect, cognition, and neurovegetative functions (American Psychiatric Association, 2013). It is one of the most frequent and impairing disorders worldwide, with an enormous impact on the individual's quality of life and a leading cause of disease burden (Ferrari et al., 2013). Most affected individuals experience their first episode during adolescence (Thapar, Eyre, Patel, & Brent, 2022) with a high risk for further depressive episodes in adulthood (Johnson, Dupuis, Piche, Clayborne, & Colman, 2018), thus early recognition and effective intervention are crucial. Still, research has focused on adult MDD for many decades; only since the late 20th century have youth been studied more intensively (Fergusson & Woodward, 2002; Maughan, Collishaw, & Stringaris, 2013). MDD prevalence has been rising during past years, even before the COVID‐19 pandemic (Thapar et al., 2022), with a particularly more rapid increase rate in youth compared to older age groups (Weinberger et al., 2018). MDD in youth is accompanied by a high risk of serious psychosocial, academic, and physical impairments, such as social withdrawal, school drop‐out, substance misuse, and obesity (Fergusson & Woodward, 2002; Thapar, Collishaw, Pine, & Thapar, 2012). Risk of suicide—a leading cause of death in youth worldwide (Glenn et al., 2020)—is also strongly increased with more than half of adolescent suicide victims having suffered from MDD (Gould, Greenberg, Velting, & Shaffer, 2003; Pelkonen & Marttunen, 2003). The lack of studies in youth with MDD contrasts strongly with this high burden (Maughan et al., 2013).

While rates of MDD are rather low (<1%) and equally distributed across sex in childhood, there is a sharp rise throughout adolescence (1‐year‐prevalence around 8%) with girls affected twice as often and experiencing more severe episodes than boys (Shorey, Ng, & Wong, 2022; Thapar et al., 2022). This “gender gap” has been suggested to be one of the most robust findings in MDD research, arising in early youth, increasing throughout adolescence, and persisting across the lifespan, even across cultures (Salk, Hyde, & Abramson, 2017); however, its underlying factors remain unknown (Thapar et al., 2022). Adolescence with its rapid neurobiological, emotional, cognitive, and psychological changes constitutes a particularly relevant time to understand pathomechanisms underlying MDD. Given the coincidence with puberty onset (Salk et al., 2017), pubertal processes and their neuroendocrine transitions (e.g., of sex hormones) have been considered to put youth, particularly girls, at higher risk for MDD (Stumper & Alloy, 2023). However, MDD prevalence in boys also increases during adolescence (Daly, 2022; Skovlund, Kessing, & Mørch, 2017), with boys showing higher suicide rates than girls (Glenn et al., 2020). Thus, it is important to also identify and prevent MDD in boys; yet, males are still overlooked and underrepresented in MDD research (Knox et al., 2023; Shi et al., 2021).

Besides the pubertal transition, exposure to psychosocial stress has been reported as one of the strongest risk factors for MDD in youth (Thapar et al., 2012). Aiming to understand the neurobiological effects of stress in MDD, previous work focused on hypothalamic–pituitary–adrenal (HPA) axis activity, which is essential for responding to and regulating the impact of stress. Salivary cortisol constitutes a widely used peripheral marker of HPA axis functioning. Attenuated salivary cortisol stress responsivity has been repeatedly reported in adults with MDD (Rothe, Steffen, Penz, Kirschbaum, & Walther, 2020). Contrastingly, results in youth with MDD are less consistent, showing both increased and decreased cortisol stress response, and lack the inclusion of possible confounding factors (e.g., puberty, body mass index [BMI], substance use, comorbid mental disorders) (Bernhard, Mayer, Fann, & Freitag, 2021). Some studies report differences between girls and boys with MDD (Mazurka, Wynne‐Edwards, & Harkness, 2018); yet, sex differences have rarely been systematically investigated (Bernhard et al., 2021). Additional research is necessary to disentangle the effects of sex on the neuroendocrine stress response in youth with MDD.

Previous research on the neuroendocrinological stress response in MDD has focused on cortisol. This approach neglects the neuroendocrine interplay during stress: the HPA axis is assumed to operate in a coordinated manner with the hypothalamic–pituitary–gonadal (HPG) axis to allow successful stress regulation (Oyola & Handa, 2017; Turan, Tackett, Lechtreck, & Browning, 2015). Sex hormones (such as testosterone) have been suggested to dampen the effects of stress, thereby showing protective effects against depression (McHenry, Carrier, Hiull, & Kabbaj, 2014). In addition, neuropeptides (such as oxytocin) are also involved in stress regulation (Carter & Kingsbury, 2022; Takayanagi & Onaka, 2022). Most stressors that stimulate HPA axis functioning also activate oxytocin, which peaks earlier than cortisol in response to stress (Bernhard et al., 2018). Oxytocin has also been reported to show antidepressant effects (Neumann & Landgraf, 2012). Yet, testosterone and oxytocin have not been investigated in response to stress in youth with MDD.

More recently, there has been a growing awareness that the different neuroendocrine systems do not operate independently but rather interact with each other in a complex manner (Sollberger & Ehlert, 2016). Particularly, the adrenal and gonadal interplay and irregularities in cross‐talk between sex steroids and glucocorticoids have been suggested to contribute to dysregulated stress responses and sex‐specific differences in the characteristics of stress‐related mental disorders (Oyola & Handa, 2017). To ideally maintain homeostasis, the neuroendocrine system continuously monitors levels of steroids. However, as HPA and HPG axes are suggested to work in parallel, dysregulation of one or both of these axes may result in an overall altered neuroendocrine system, thereby influencing altered neuroendocrine responses to stressful live events (Oyola & Handa, 2017). Consequently, rather than assessing a single hormonal measure only, a dual‐axis approach has been suggested to better reflect the neuroendocrine complexity by assessing multiple hormones and their coordination (Shirtcliff et al., 2015; Turan et al., 2015). However, examining more than one hormone particularly in response to stress has not been addressed in adolescents with MDD to date.

Taken together, the current study aimed at comprehensively investigating multiple neuroendocrine systems (HPA axis: cortisol, HPG axis: testosterone, neuropeptides: oxytocin) in response to a standardized psychosocial stress test in girls and boys with MDD compared to healthy adolescents. Given previous evidence of cortisol hypo‐reactivity in adults with MDD, and assumed parallel activation of the HPA axis, HPG axis, and neuropeptide system in response to stress, we hypothesized an overall attenuated neuroendocrine stress response in MDD compared to healthy controls (HCs). Given the sex‐specific findings in the onset of adolescent MDD, we expect a stronger attenuated neuroendocrine stress response in girls than boys with MDD.

## Methods

### Participants

This study included 103 pubertal youth (12–18 years) with MDD (62.1% females) and 72 HCs (62.5% females). Data were collected between 2014 and 2018 at the Goethe University Hospital Frankfurt am Main, Germany, with the approval of the local ethical committee. Adolescents with MDD were recruited from the clinic, and HCs from the community within the Frankfurt part of the FemNAT‐CD study (Freitag et al., 2018). Written informed consent was obtained from all participants and their legal guardians after study explanation. Exclusion criteria included IQ <70, pre‐pubertal status, pregnancy, last menstruation >6 months, history of neurological disorder, traumatic brain injury, schizophrenia, autism spectrum disorder, disruptive behavior disorders, and current mania or bipolar disorder. Youth with MDD fulfilled the current clinical DSM‐IV‐TR MDD diagnosis, while HCs were free of any current DSM‐IV‐TR disorder.

### Procedures

Current and lifetime mental disorders were assessed with the Kiddie‐Schedule for Affective Disorders and Schizophrenia‐Present and Lifetime version (K‐SADS‐PL; Kaufman et al., 1997), IQ by the Wechsler Intelligence Scales (Wechsler, 2003, 2008), and pubertal status using the Pubertal Development Scale (Petersen, Crockett, Richards, & Boxer, 1988). BMI was calculated based on measured weight and height, and medication and smoking assessed via self‐reports. For details of all measures (see Appendix S1).

### Psychoneuroendocrine assessment

**Figure 1:**
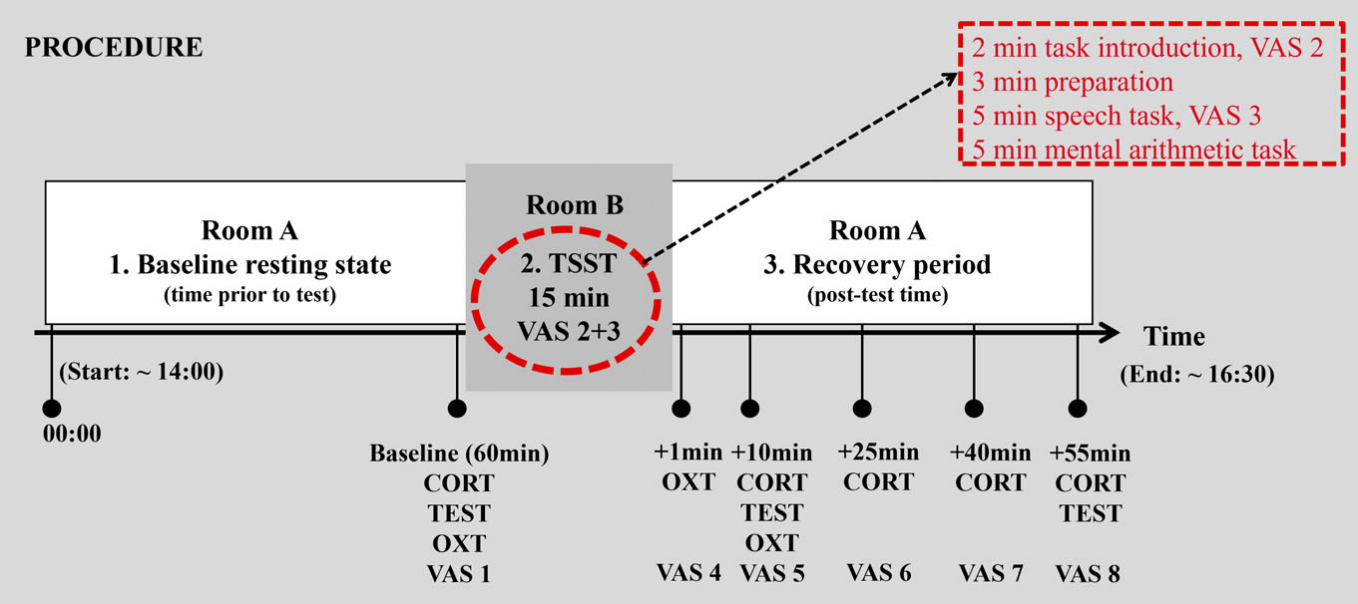
Assessment of psychoneuroendocrine stress response. After a relaxation period in a comfortable room (Room A), the baseline assessment was taken before participants entered a sparsely equipped experimental room (Room B). The Trier Social Stress Test (TSST) involved four components: task introduction, preparation time, speech task, and an age‐adapted mental arithmetic task. CORT, cortisol; OXT, oxytocin; TEST, testosterone; VAS, Visual Analogue Scale

Stress responsivity was assessed using the Trier Social Stress Test (TSST; Kirschbaum, Pirke, & Hellhammer, 1993), a widely used, standardized method for inducing psychosocial stress in laboratory settings (Allen, Kennedy, Cryan, Dinan, & Clarke, 2014). The TSST involves completing public speaking and mental arithmetic tasks in front of a panel of strangers (see Figure 1, Appendix S2). To confirm stress induction, participants rated their stress feelings (“Do you feel stressed?”, 0 = *no, not at all* to 10 = *yes, very much*) eight times from baseline to +55 min after stress termination using a Visual Analogue Scale (range 0–10) (Hellhammer & Schubert, 2012). Saliva samples were collected by Salivettes (Sarstedt, Germany) at baseline, +1, +10, +25, +40, and +55 min after stress termination. Samples were stored at −20°C until analysis.

Levels of salivary cortisol (nmol/L) at baseline, +10, +25, +40, and +55 min, and testosterone (pg/mL) at baseline, +10, and +55 min were analyzed by daacro's Saliva Lab (Trier, Germany) employing an enzyme immunoassay kit (Salimetrics, LLC; Carlsbad, USA) formatted to minimize cross‐reactivity for related steroids. The corresponding inter‐ and intra‐assay coefficients of variation were <15% and <10%, respectively (Salimetrics, 2024). After cortisol and testosterone analyses, the baseline, +1, and +10 min Salivettes were transferred to RIAgnosis (Sinzing, Germany) for quantification of salivary oxytocin (pg/mL) by radioimmunoassay. The detection limit was in the 0.1–0.5 pg/sample range. Intra‐ and inter‐assay coefficients of variation were <10%. Oxytocin was measured in a subsample of 143 participants (see Table S1), including 93 youth with MDD (62.4% females) and 50 HCs (56.0% females). Collection of samples at the +1 min timepoint started around 1 year into the study, after validation of salivary oxytocin stress responsivity in youth (Bernhard et al., 2018). Different timepoints for cortisol, testosterone, and oxytocin analyses relate to distinct reactivity patterns (Allen et al., 2014; Bernhard et al., 2018; de Jong et al., 2015).

### Statistical analyses

Statistical analyses are based on SPSS v29 (IBM Corp., Armonk, NY) and R v4.2.0. Neuroendocrine measures were log‐transformed, dimensional covariates mean‐centered (Delaney & Maxwell, 1981), and significance levels set at *p* ≤ .05 (two‐tailed). Demographic and clinical characteristics were compared using univariate analyses of variance or chi‐squared tests. To investigate the neuroendocrine interplay of the HPA axis, HPG axis, and neuropeptide system, correlations between the area under the curve with respect to increase (AUC_I_; i.e., with reference to the baseline value) of the different psychoneuroendocrine measures (Pruessner, Kirschbaum, Meinlschmid, & Hellhammer, 2003) were calculated using Spearman correlations.

Psychoneuroendocrine stress response was analyzed using four separate hierarchical linear models (HLM; lm4 package). The four dependent variables were psychological stress, cortisol, testosterone, and oxytocin, with the independent variable of group (MDD vs. HCs) interacting with a linear (polynomial 1; i.e., increase) and quadratic (polynomial 2; i.e., increase and decrease) time effect. Along with the individual participant as a random effect (within‐person effects), analyses were controlled for sex, age, pubertal status, BMI, and current smoking (psychological stress analyses: age and pubertal status only) as fixed effects. To investigate differences between girls and boys, sex was additionally included as an independent variable to investigate three‐way interactions between group (MDD vs. HCs), sex (female vs. male), and time (polynomial 1 and polynomial 2). To follow up possible interactions of sex, group, and any time factor, each HLM was repeated in female and male youths separately. To test for possible influences of comorbid mental disorders and medication in sensitivity analyses (in case of significant descriptive group differences), each HLM was repeated including one of the following potential confounding factors: attention‐deficit/hyperactivity disorder (ADHD), anxiety disorders (ANX), posttraumatic stress disorder (PTSD), eating disorders (EAT), selective serotonin reuptake inhibitor SSRIs/antidepressant medication, and other medication. Besides the strengths of the HLM approach (Dijkers, 2013), repeated measures analyses of covariance (rmANCOVA) were also performed (see Appendix S3, Table S5) for easier comparison with previous work (Bernhard et al., 2021).

## Results

### Sample characteristics

MDD and HC groups did not differ in sex, age, pubertal status, IQ, parental educational status, or TSST start time (see Table 1; data for oxytocin subsample Table S1). Participants with MDD had higher BMIs and rates of current smoking, medication use (antipsychotics, SSRIs/antidepressants, other), and comorbid mental disorders (ADHD, ANX, PTSD, EAT). Most female participants had started menstruating (MDD 98%, HCs 91%) with comparable days since the last menstruation (Mean [*SD*]: MDD 17.78 [8.43], HCs 16.98 [9.32], *p* = .67; 88% complete information).

**Table 1:** Sample description in participants with major depressive disorder (MDD) compared to healthy controls (HCs)

	Females (*n* = 109)		Males (*n* = 66)		Group	Sex	Group × Sex
	MDD (*n* = 64)	HCs (*n* = 45)	MDD (*n* = 39)	HCs (*n* = 27)			
	Mean (*SD*) or *n* (%)				*p*	*p*	*p*
Age (years)	15.22 (1.5)	15.00 (1.8)	15.49 (1.4)	15.00 (1.8)	.15	.58	.58
Estimated full‐scale IQ	106.29 (10.7)	109.11 (12.1)	106.47 (9.24)	102.87 (12.1)	.82	.08	.07
Parental educational status^a^	3.70 (1.1)	4.26 (0.9)	3.41 (1.0)	3.39 (0.8)	.08	<.001	.06
Body mass index	23.46 (5.1)	20.68 (3.5)	24.54 (5.7)	21.11 (3.1)	<.001	.13	.97
Start time Trier Social Stress Test (hr:min)	15:15 (00:23)	15:13 (00:32)	15:08 (00:28)	15:14 (00:27)	.29	.20	.11
Pubertal status^a^					.10	<.001	
Mid‐pubertal	1 (1.6)	4 (8.9)	13 (27.1)	15 (55.6)			
Late‐pubertal	44 (68.8)	30 (66.7)	25 (47.9)	12 (44.4)			
Post‐pubertal	19 (29.7)	11 (24.4)	1 (2.6)	0 (0.0)			
Current smoking	27 (42.2)	2 (4.4)	9 (23.1)	3 (11.1)	<.001	.20	
Any medication	51 (79.7)	13 (20.3)	30 (76.9)	3 (11.1)	<.001	.60	
Antipsychotics	2 (3.1)	0 (0.0)	0 (0.0)	0 (0.0)	.02	.04	
Stimulants	12 (14.6)	0 (0.0)	1 (2.6)	0 (0.0)	.14	.88	
Non‐stimulants^b^	0 (0.0)	0 (0.0)	1 (2.6)	0 (0.0)	.40	.20	
SSRIs/antidepressants	45 (70.3)	0 (0.0)	26 (66.7)	0 (0.0)	<.001	.81	
Tranquilizer	2 (3.1)	0 (0.0)	0 (0.0)	0 (0.0)	.23	.27	
Contraceptives	10 (16.4)	6 (15.4)	0 (0.0)	0 (0.0)	.89		
Other	18 (28.1)	5 (11.1)	12 (30.8)	3 (11.1)	<.01	.80	
Comorbid mental disorders							
Lifetime ADHD	5 (7.8)	0 (0.0)	4 (10.3)	0 (0.0)	.01	.67	
Lifetime substance use disorder	0 (0.0)	0 (0.0)	4 (10.3)	0 (0.0)	.09	<.01	
Lifetime anxiety disorder^c^	30 (46.9)	2 (4.4)	20 (51.3)	2 (3.6)	<.001	.89	
Lifetime PTSD	9 (14.1)	1 (2.2)	2 (5.1)	1 (3.7)	.05	.26	
Lifetime eating disorder	13 (12.6)	0 (0.0)	1 (1.0)	0 (0.0)	<.001	.01	
Baseline psychoneuroendocrine levels							
Psychological stress	3.05 (3.1)	1.00 (1.6)	2.83 (2.6)	0.80 (1.3)	<.001	.59	.98
Cortisol (nmol/L)	3.66 (1.8)	3.28 (2.1)	3.31 (1.7)	3.03 (1.5)	.25	.28	.86
Testosterone (pg/mL)	45.55 (18.1)	33.85 (13.4)	91.4 (43.8)	58.47 (29.2)	<.001	<.001	.01
Oxytocin (pg/mL)	1.33 (0.3)	1.57 (0.8)	1.27 (0.3)	1.17 (0.8)	.50	.02	.09

ADHD, attention‐deficit/hyperactivity disorder; PTSD, posttraumatic stress disorder; SSRI, selective serotonin reuptake inhibitor/antidepressant medication.

^a^ For definition of parental educational status and pubertal categories, see Appendix S1.

^b^ Non‐stimulants include atomoxetine medication ‘Other’ includes asthma medication, painkiller, or vitamin preparation. Participants may have been prescribed more than one medication category.

^c^ Rates of lifetime anxiety disorder cover lifetime panic disorder, separation anxiety disorder, avoidant disorder, simple phobia, social phobia, agoraphobia, and overanxious disorder/generalized anxiety disorder.

### Baseline levels

Youth with MDD reported higher psychological stress (mean [*SD*]: MDD = 2.97 [2.90], HCs = 0.93 [1.51], *p* < .001) and showed higher testosterone levels (pg/mL; mean [*SD*]: MDD = 62.79 [37.55], HCs = 43.08 [23.80], *p* < .001) at baseline compared to HCs. Baseline cortisol (nmol/L; mean [*SD*]: MDD = 3.53 [1.73], HCs = 3.19 [1.85], *p* = .25) and oxytocin (pg/mL; mean [*SD*]: MDD = 1.31 [0.29], HCs = 1.42 [0.83], *p* = .50) did not differ between groups. Boys showed higher levels of baseline testosterone than girls (pg/mL; mean [*SD*]: girls = 40.67 [17.25], boys = 77.71 [41.47], *p* < .001) with particularly high levels in males with MDD (significant group‐by‐sex effect; see Table S1). Girls showed higher levels of baseline oxytocin than boys (pg/mL; mean [*SD*]: girls = 1.38 [0.77], HCs = 1.31 [0.29], *p* = .02), irrespective of group status. No other sex or group‐by‐sex effects were found for baseline psychoneuroendocrine levels (see Table 1).

### Correlations between psychoneuroendocrine stress responses

AUC_I_ of cortisol and testosterone correlated positively in youth with MDD (*n* = 98, *r* = .42, *p* < .001), HCs (*n* = 67, *r* = .43, *p* < .001), and the overall sample (*n* = 165, *r* = .44, *p* < .001). A moderate to strong positive correlation was found in females for all groups (MDD, HCs, overall), for males only in the overall sample. No significant correlations with oxytocin nor of psychological stress with any of the neuroendocrine measures were found (see Table S3).

### Hierarchical linear models of psychoneuroendocrine stress response

**Figure 2:**
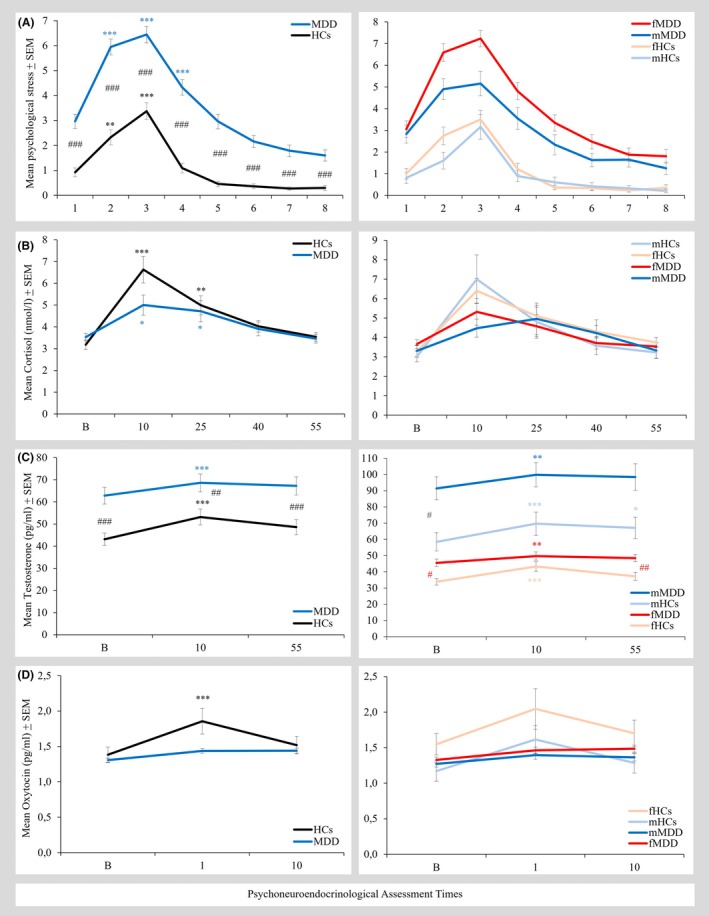
Psychoneuroendocrine stress response during the Trier Social Stress Test in participants with major depressive disorder (MDD) compared to healthy controls (HCs). Psychological stress (A), cortisol (B), testosterone (C), and oxytocin (D) responses to psychosocial stress in the overall (left) and sex‐separated groups (right). Significant difference with corresponding basal value: *p < .05, **p < .01, ***p < .001. Significant group difference: ^#^ *p* < 0.05, ^##^ *p* < 0.01, ^###^ *p* < 0.001. fHCs, female HCs; fMDD, female MDD; mHCs, male HCs; mMDD, male MDD

Results of HLM are shown in Table 2. Figure 2 and Table S2 present raw data; Figure S1 shows log‐transformed data. Psychological stress was successfully induced in MDD and HCs (main effect of linear and quadratic change in time). Individuals with MDD showed higher psychological stress than HCs (main effect of group) and a stronger linear stress response (interaction of group‐by‐time) which was evident in both females and males with MDD (no interaction effects with sex; same in sex‐specific analyses).

**Table 2:** Results of hierarchical linear models (HLM) of psychoneuroendocrine stress responses in participants with major depressive disorder (MDD) compared to healthy controls (HCs)

	Psychological stress				Cortisol				Testosterone				Oxytocin			
	*b*	*SE*	*β*	*p*	*b*	*SE*	*β*	*p*	*b*	*SE*	*β*	*p*	*b*	*SE*	*β*	*p*
Overall (*N* = 175)																
Age	0.05	0.05	0.05	.37	0.14	0.08	0.14	.07	0.11	0.07	0.11	.11	−0.01	0.09	−0.01	.89
Pubertal status	0.05	0.06	0.05	.41	0.10	0.09	0.10	.27	0.10	0.08	0.10	.21	−0.01	0.11	−0.01	.94
Sex^a^	0.17	0.12	0.08	.14	0.05	0.17	0.02	.76	−1.15	0.15	−0.56	**<.01**	0.30	0.20	0.14	.14
Group	0.75	0.09	0.37	**<.01**	−0.04	0.14	−0.02	.79	0.44	0.13	0.22	**<.01**	−0.07	0.17	−0.03	.68
Time (poly 1)	−8.28	1.01	−0.22	**<.01**	0.48	0.86	0.02	.58	1.12	0.49	0.05	.**02**	3.20	1.33	0.14	.**02**
Time (poly 2)	1.97	1.01	0.05	.**05**	−7.95	0.86	−0.27	**<.01**	−3.13	0.49	−0.14	**<.01**	−5.88	1.33	−0.26	**<.01**
Group × Time (poly 1)	−7.62	1.32	−0.16	**<.01**	−1.68	1.13	−0.04	.14	−0.24	0.65	−0.01	.71	−0.87	1.60	−0.03	.59
Group × Time (poly 2)	−1.15	1.32	−0.02	.38	3.84	1.13	0.10	**<.01**	1.75	0.64	0.06	.**01**	5.01	1.60	0.18	**<.01**
Sex^b^	<0.01	0.16	<0.01	.98	0.07	0.23	0.03	.76	−1.01	0.21	−0.49	**<.01**	0.65	0.30	0.31	.**03**
Group × Sex	0.28	0.19	0.13	.14	−0.03	0.27	−0.02	.90	−0.23	0.24	−0.11	.34	−0.5	0.32	−0.25	.12
Sex × Time (poly 1)	−2.67	2.08	−0.06	.20	1.57	1.78	0.04	.38	−1.39	1.02	−0.05	.17	−3.63	2.75	−0.13	.19
Sex × Time (poly 2)	2.45	2.08	0.05	.24	−0.99	1.78	−0.03	.58	−1.53	1.01	−0.05	.13	5.62	2.75	0.19	.**04**
Group × Sex × Time (poly 1)	−0.7	2.73	−0.01	.80	−2.16	2.33	−0.04	.35	1.89	1.33	0.05	.16	4.57	3.31	0.13	.17
Group × Sex × Time (poly 2)	−3.73	2.72	−0.06	.17	3.91	2.33	0.08	.09	1.73	1.33	0.05	.19	−5.22	3.31	−0.15	.11
Females (*n* = 109)																
Age	0.10	0.07	0.09	.15	0.14	0.09	0.16	.10	0.04	0.08	0.05	.66	−0.05	0.10	−0.06	.60
Pubertal status	−0.04	0.08	−0.04	.59	0.07	0.11	0.06	.52	−0.02	0.10	−0.02	.84	0.08	0.13	0.07	.51
Group	0.87	0.12	0.41	**<.01**	0.04	0.17	0.02	.80	0.40	0.16	0.25	.**01**	−0.24	0.21	−0.12	.24
Time (poly 1)	−9.27	1.36	−0.23	**<.01**	1.08	1.08	0.04	.32	0.60	0.67	0.03	.36	1.90	1.7	0.09	.26
Time (poly 2)	2.89	1.36	0.07	.**03**	−8.32	1.08	−0.30	**<.01**	−3.70	0.66	−0.20	**<.01**	−3.87	1.7	−0.17	.**02**
Group × Time (poly 1)	−7.89	1.78	−0.15	**<.01**	−2.50	1.41	−0.07	.08	0.47	0.87	0.02	.59	0.78	2.06	0.03	.70
Group × Time (poly 2)	−2.54	1.77	−0.05	.15	5.30	1.41	0.15	**<.01**	2.40	0.86	0.10	.**01**	3.15	2.06	0.12	.13
Males (*n* = 66)																
Age	−0.05	0.09	−0.05	.58	0.13	0.16	0.11	.45	0.27	0.13	0.27	.**04**	0.09	0.20	0.08	.64
Pubertal status	0.24	0.10	0.24	.**02**	0.19	0.18	0.15	.28	0.27	0.14	0.25	.**05**	−0.19	0.22	−0.16	.39
Group	0.53	0.14	0.30	**<.01**	−0.16	0.25	−0.07	.54	0.41	0.20	0.21	.**04**	0.28	0.29	0.13	.34
Time (poly 1)	−6.61	1.45	−0.20	**<.01**	−0.50	1.44	−0.02	.73	1.98	0.71	0.09	.**01**	5.53	2.08	0.24	.**01**
Time (poly 2)	0.44	1.45	0.01	.76	−7.33	1.44	−0.23	**<.01**	−2.17	0.71	−0.10	**<.01**	−9.49	2.08	−0.41	**<.01**
Group × Time (poly 1)	−7.20	1.90	−0.17	**<.01**	−0.33	1.88	−0.01	.86	−1.41	0.93	−0.05	.13	−3.79	2.48	−0.14	.13
Group × Time (poly 2)	1.20	1.89	0.03	.53	1.40	1.88	0.03	.46	0.67	0.93	0.02	.47	8.37	2.48	0.30	**<.01**

Time (poly 1) represents a linear (polynomial 1), and time (poly 2) a quadratic (polynomial 2) time effect. Reference category for sex = female, and for group = MDD. SE = Standard error of *b*. Significant effects are displayed in bold: *p < .05, **p < .01, ***p < .001.

^a^ Sex included as a fixed effect in HLM.

^b^ Sex included as an additional independent variable to test for three‐way interaction effects of group, sex, and time in HLM (see also Methods).

The TSST also induced a neuroendocrine stress response for cortisol, testosterone, and oxytocin in both participants with MDD and HCs (for all main effects of quadratic change in time, which were also evident in females and males separately). Main effects of group and sex indicated generally higher testosterone levels in youth with MDD than HCs (also in sex‐specific models), and in males compared to females. Furthermore, both females and males with MDD showed a lower cortisol, testosterone, and oxytocin stress response than HCs (interaction of group‐by‐time for quadratic changes in time; no interaction effects with sex). In sex‐specific HLMs, effects of group‐by‐time (quadratic changes in time) for cortisol and testosterone were found in females, while they appeared for oxytocin in males. Classical rmANCOVAs showed similar results (see Table S5), with a stronger testosterone group‐by‐time interaction effect in HLM analyses.

### Sensitivity analyses

When repeating each HLM considering possible effects of comorbid mental disorders (ADHD, ANX, PTSD, EAT) and medication (SSRI, other), effects for all psychoneuroendocrine measures remained unchanged (see Table S4).

## Discussion

This is the largest study to date investigating psychoneuroendocrinological stress responsivity in youth with MDD compared to HCs thereby considering multiple neuroendocrine axes. Our results considerably extend previous studies on cortisol stress response in adolescents with MDD by providing evidence for an altered neuroendocrine stress responsivity not only of the HPA axis but also of the HPG axis and neuropeptide system in youth with MDD. Remarkably, parallel to increased acute psychological stress, neuroendocrine hypo‐reactivity was observed in both girls and boys with MDD compared to healthy youth.

The TSST induced a distinct increase of subjectively experienced psychological stress in both MDD and HC participants, demonstrating the applied procedure to be effective in inducing stress. Corroborating social stress as a risk for MDD in youth (Thapar et al., 2012), psychological stress responsivity was strongly elevated in girls and boys with MDD compared to HC. Girls and boys with MDD reported higher baseline psychological stress prior to the TSST and showed even stronger increases in their subjective stress level in response to acute psychosocial stress. Results are in line with previous findings of increased experienced stress‐related negative thoughts (Stadelmann et al., 2018) and expressed stress (Klimes‐Dougan et al., 2019) in response to the TSST in youth with MDD. Particularly, the combination of increased experienced and expressed psychological stress, but decreased physiological (i.e., cortisol) response to the TSST was found to predict depressive symptoms in adolescents with and without MDD (Bendezú, Thai, Wiglesworth, Cullen, & Klimes‐Dougan, 2022; Carosella et al., 2023). However, these studies were over‐sampled for adolescent girls showing non‐suicidal self‐injury (NSSI); thus, future work should disentangle the influence of this commonly reported behavior in adolescents (Plener et al., 2018). Besides, the discrepant psychological and physiological stress response was not only found in our study (see below) but also in adolescent anxiety disorders (Krämer et al., 2012; Stadelmann et al., 2018). Comorbid anxiety disorders were highly prevalent in our sample as previously reported in youth with MDD (Kalin, 2020); yet, findings remained stable when including comorbid anxiety disorders in sensitivity analyses. In contrast, youth with disruptive behavior disorders showed no differences in their experienced psychological stress response compared to HCs (Bernhard et al., 2022; Fairchild, Baker, & Eaton, 2018). The discrepancy between psychological and neuroendocrine stress responsivity in adolescents with MDD may thus be a specific characteristic related to internalizing rather than externalizing disorders. Though previously rather neglected, the combined assessment of psychological and physiological stress response measures is thus important to increase information about an individual's risk for stress‐related psychopathology (Bendezú et al., 2022; Campbell & Ehlert, 2012).

Furthermore, clear cortisol, testosterone, and oxytocin responses to the TSST were observed in both MDD and HCs; for the latter comparable to those previously reported (Allen et al., 2014; Bernhard et al., 2018; Turan et al., 2015). As expected, attenuated cortisol stress responsivity was found in youth with MDD compared to HCs, in line with earlier reports (Harkness, Stewart, & Wynne‐Edwards, 2011; Klimes‐Dougan et al., 2019; Stadelmann et al., 2018). Results were robust when including comorbid mental disorders or antidepressant medication in sensitivity analyses, in line with findings in adults with MDD showing no influence of pharmacotherapy on HPA axis functioning (McKay & Zakzanis, 2010). Notably, in this so far largest sample on youth with MDD, we found cortisol hypo‐reactivity in both female and male youth with MDD (no interaction of group, sex, and time), in line with Stadelmann et al. (2018). However, other earlier work in youth and adults with MDD reported elevated cortisol stress responses in males compared to females (Mazurka et al., 2018; Zorn et al., 2017). Yet, they did not consider the influence of potentially confounding variables (particularly age), and females and males were not compared within one sample.

Extending previous work limited to cortisol, our study additionally investigates testosterone and oxytocin stress response in adolescents with MDD. We observed increased testosterone, independent of the stress‐inducing procedure, in females and males with MDD compared to HCs, in line with previous reports suggesting increased testosterone as a risk factor for MDD particularly in youth (Chronister et al., 2021; Copeland, Worthman, Shanahan, Costello, & Angold, 2019; Yin et al., 2024). Higher levels of testosterone have also been found in adult females with severe MDD (Weber, Lewicka, Deuschle, Colla, & Heuser, 2000). Yet, it contradicts findings (Culbert et al., 2022; Granger et al., 2003) of attenuated testosterone levels in boys with depressive symptoms (but not clinical MDD diagnosis), and studies on predominantly male adults, in which antidepressant effects of testosterone have been discussed (Anderson et al., 2022). Conflicting male adolescent (Duke, Balzer, & Steinbeck, 2014) and adult (Zito, Nosari, Pigoni, Moltrasio, & Delvecchio, 2023) findings on testosterone and mood disorders may be influenced by high study heterogeneity (e.g., age, MDD severity, pubertal status, assay and sampling methods, psychometric scale use). Particularly, age and clinical diagnosis of MDD seem to play an important role. Findings of antidepressant effects of testosterone are limited to rather older male adults, and only in relation to depressive symptoms rather than clinical diagnosis of MDD (Anderson et al., 2022; Vartolomei, Kimura, Vartolomei, & Shariat, 2020). Furthermore, in line with our hypotheses, attenuated testosterone stress response was found in MDD compared to HCs, which was evident in both female and male adolescents (no interaction of group, sex, and time). Our results also show cortisol and testosterone to be strongly positively correlated, in line with the previously reported coupling of stress and sex steroids (King, Graber, Colich, & Gotlib, 2020). Hence, the blunted cortisol and testosterone stress response found in MDD suggests an overall altered steroid system in youth with MDD compared to HCs in response to acute stress. However, as previous studies investigating testosterone stress response in individuals with MDD are missing, future studies are needed to replicate our findings of attenuated testosterone stress response in adolescents with MDD.

For oxytocin, our results indicate an attenuated stress response in female and male youth with MDD compared to HCs as hypothesized. While oxytocin reactivity to psychosocial stress has been reported in healthy adolescents (Bernhard et al., 2018) and adults (Alley, Diamond, Lipschitz, & Grewen, 2019; de Jong et al., 2015; Engert, Koester, Riepenhausen, & Singer, 2016; Pierrehumbert et al., 2010; Tabak et al., 2022), this study advances previous work by assessing the oxytocin stress response also in individuals with MDD. Blunted oxytocin stress response has been found in adolescents with conduct disorder (Bernhard et al., 2022) and adults with borderline personality disorder (Aboulafia‐Brakha et al., 2023), while oxytocin reactivity in youth with anxiety disorders is yet to be studied (Goetz et al., 2021). Overall, studies investigating oxytocin response to psychosocial stress in individuals affected by mental disorders are still rare. This is rather surprising, given the reported significant effects of oxytocin in stress regulation and psychopathology (Takayanagi & Onaka, 2022). Understanding the influence of oxytocin on stress‐related disorders such as MDD may play an important role for prevention and intervention strategies. Indeed, higher oxytocin reactivity has recently been related to greater treatment response and symptom reduction in adults with MDD, supporting the oxytocin system as an important biomarker and moderator of treatment success (Atzil‐Slonim et al., 2022). Thus, more work is needed to investigate possible effects of oxytocin on stress regulation in MDD.

Taken together, our findings of attenuated steroid and neuropeptide stress response suggest an overall altered neuroendocrine stress regulation in youth with MDD. While previous work in adolescents assessing multiple neuroendocrine (such as of cortisol, testosterone, and/or oxytocin) stress responses is scarce, previous reports indicate the coordinated stimulation of the HPA and HPG axes in response to acute stress in healthy adults (Domes, Linnig, & von Dawans, 2024) and adolescents (Rodgers & Kuhlman, 2023). To follow this idea, within‐person coupling or latent profile analyses (Howland, Donzella, Miller, & Gunnar, 2020; Marceau et al., 2013) may be more appropriate to investigate neuroendocrine coordination rather than separate neuroendocrine statistical models, ideally in larger samples of multiple neuroendocrine measures with concurrent assessment times. However, our results of overall blunted cortisol, testosterone, and oxytocin stress response in adolescents with MDD compared to HC's and the positive correlations between cortisol and testosterone, may be in line with the idea of a coordinated functional stress responsiveness of different neuroendocrine systems. This may be relevant for short‐ and long‐term stress regulation (e.g., cortisol activating energy resources, testosterone improving performance, oxytocin with anxiolytic effects) (Oyola & Handa, 2017; Turan et al., 2015). Dysregulated neuroendocrine stress responsivity may follow chronic stress exposure, resulting in an overall blunted and less responsive neuroendocrinological stress system to avoid allostatic overload, thereby increasing risk for stress‐related psychopathology (Del Giudice, Ellis, & Shirtcliff, 2011; Holochwost et al., 2021). Future work should thus consider the influence of adverse events, particularly during the course of neuroendocrine activation in the trajectory of depressive symptoms (Peckins, Negriff, Gordis, Zhen, & Susman, 2024). Further studies, ideally longitudinal, studying neuroendocrine characteristics and associated risk factors in the course of MDD (i.e., before onset, during full episode, after remission), and across different age groups are needed to investigate whether neuroendocrine alterations in response to stress may change over time and with clinical symptomatology. If replicated, our findings of a possibly fundamentally changed psychoneuroendocrinological stress response in youths with MDD may carry important clinical implications. Blunted neuroendocrine support (implemented by limited energy resources, performance support, and anxiolytic effects via attenuated cortisol, testosterone, and oxytocin contribution) in stress conditions may biologically hinder successful management of stressful situations in individuals vulnerable for stronger stress experiences, thereby possibly increasing depressive symptomatology. This idea is supported by findings of altered neuroendocrine stress regulation in youth with MDD increasing vulnerability for suicide in acute stress situations (Miller & Prinstein, 2019). Given previous reports on functional change of the neuroendocrine stress system (e.g., greater cortisol and oxytocin reactivity) during treatment response in adults with depressive symptoms (Atzil‐Slonim et al., 2022; Roque et al., 2020), treatment effects on neuroendocrine stress response alterations in youth with MDD should also be investigated in future work. Considering individuals' psychoneuroendocrine characteristics in personalized treatments, for example, by studying pharmacological treatments to normalize neuroendocrine hypo‐reactivity, may also improve successful interventions for MDD. However, studies investigating pharmacological treatments in affected or at‐risk individuals to normalize neuroendocrine hypo‐reactivity are lacking.

In contrast to our expectations and the reported ‘gender gap’ in MDD (Salk et al., 2017), we did not find the blunted steroid and neuropeptide stress responses to be influenced by sex (as indicated by missing interaction effects of group, sex, and time). When analyzing females and males separately, linear (i.e., increase) as well as quadratic (i.e., increase and decrease) changes in time were found for all neuroendocrine measures in both females and males separately. However, the attenuated cortisol and testosterone stress responses were found to be more pronounced in females, and the blunted oxytocin stress response was more evident in male adolescents. Given that the sample size differed between females (*n* = 109) and males (*n* = 66) and was overall lower for the oxytocin sample, this may have influenced the statistical power in sex‐specific analyses. Also, the descriptive characteristics between females and males are more heterogeneous (e.g., age, puberty). Given the higher prevalence of MDD in females than in males (Shorey et al., 2022), the absolute number of included boys compared to girls was subsequently lower. In the future, further (larger) samples should include more similar rates of females and males with MDD. However, the female:male ratio was balanced across groups (62% female, respectively) and sex included either as a fixed effect or an independent variable showing no interactions with group and time while controlling for the effects of age and pubertal status. Thus, altered psychoneuroendocrine stress reactivity in adolescents may constitute a distinct characteristic of MDD independent of sex. Previous work on neuroendocrine stress response in MDD hardly considered confounding factors (e.g., age, puberty, smoking, menstrual cycle, BMI) (Bernhard et al., 2021), though these moderators have been reported to strongly influence the human stress response (Narvaez Linares, Charron, Ouimet, Labelle, & Plamondon, 2020). This may underly divergent findings of previous studies. While the ‘gender gap’ seems to be particularly relevant for MDD onset, recent work suggests it to be changing, with a decrease in more gender‐equal countries, such as Germany (Bracke, Delaruelle, Dereuddre, & Van de Velde, 2020). Interestingly, our findings of a blunted cortisol stress response irrespective of sex are in line with an earlier German study (Stadelmann et al., 2018), but not a Canadian one (Mazurka et al., 2018). While societal differences may also constitute a contributing factor to be considered in MDD, findings need to be replicated and more work on sex differences across development is warranted.

This study has several strengths. It extends previous work by simultaneously investigating different measures of psychoneuroendocrinological stress responsivity (psychological, cortisol, testosterone, oxytocin) in youth with MDD compared to healthy adolescents. We included a large, representative clinical sample of girls and boys with MDD, allowing sex‐specific analyses. Participants with MDD and HCs were diagnosed using standardized semi‐structured diagnostic interviews based on DSM‐IV‐TR criteria. The TSST as a standardized, validated, and widely used laboratory psychosocial stress procedure was implemented and compared to HCs, whose psychoneuroendocrine stress responsivity demonstrated successful stress induction. Adherence to all procedures (e.g., TSST, saliva sampling/shipping) was ensured by standard operating procedures controlled by regular internal monitoring. We also controlled for highly relevant confounders and applied additional sensitivity analyses on comorbid mental disorders and medication confirming the stability of our results.

This study has also several limitations. First, only pubertal adolescents were included given the higher MDD prevalence in this age group and much lower rates of MDD (1%–2%) in pre‐pubertal children (Maughan et al., 2013). Consequently, the number of younger participants (12–14 years, *n* = 61) compared to older participants (15–18 years, *n* = 114) included in this study was substantially lower. Considering developmental trajectories of neuroendocrine stress responsivity in children and adolescents with MDD, future studies should include more younger age groups. Although statistically not significant, boys tended to show lower pubertal status than girls which is in line with a generally delayed pubertal onset in boys. Thus, pubertal status as well as age were consistently controlled for in all statistical analyses though they did not show influence in our main analyses. Exploratory analyses (see Table S6) including group, sex, and age as independent variables indicated stability of findings irrespective of age with the exception of oxytocin showing greater stress response differences between groups in older adolescents. This may be attributable to the development of the oxytocin system during adolescence (Sannino, Chini, & Grinevich, 2017) suggesting the influence of oxytocin in stress responding to be more pronounced in older than younger adolescents with MDD. However, besides the lower rate of included younger participants, our oxytocin sample was also smaller than that of cortisol and testosterone. Given the scarce work on oxytocin in children and adolescents, more research is needed to investigate oxytocin across age. Nonetheless, our oxytocin sample is still relatively large (*n* = 143) compared to previous studies. We assessed salivary oxytocin, which has been a topic of controversy, particularly due to its assumed short half‐life, molecular weight, and impurity (McCullough, Churchland, & Mendez, 2013). On the other hand, salivary oxytocin has been consistently reported as a valid, non‐stressful assessment in youth and adults correlating with its central concentrations (Martin et al., 2018), and the range of oxytocin values was in line with previous work (Bernhard et al., 2018; de Jong et al., 2015; McCullough et al., 2013). Second, storage of saliva samples at −20°C rather than −80°C was reported as less optimal (Granger, Shirtcliff, Booth, Kivlighan, & Schwartz, 2004). However, storage was consistent across groups, and possible degradation by storage temperature would have influenced absolute, rather than relative concentrations. Third, information on menstrual cycle status was assessed via self‐reported dates of last menstruation. The female MDD and HC groups did not differ in menstrual cycle status (days since last menstruation were also uncorrelated to age and pubertal status, *p* ≥ .64) with most girls being tested in the luteal phase, during which the neuroendocrine stress response is more comparable to male individuals (Kajantie & Phillips, 2006). Thereby, we followed recent recommendations (Schmalenberger et al., 2021) for assessing menstrual cycle, for example, by consistently timing the assessment in one (here: luteal) cycle phase. However, it should be noted that results may differ with regard to other menstrual cycle phases (e.g., follicular phase). Current recommendations (Schmalenberger et al., 2021) also suggest to include only participants with typical cycle lengths. With a range of 21–36 days, participant cycle lengths slightly differed from the suggested 25–35 days, yet, only for a small sample of participants (*n* = 7). Besides, it can be useful to assess not only the date of begin of the last menstrual cycle, but also the start date of the next expected cycle to apply a backward‐count for the exact menstrual cycle phase on the day of outcome assessment. Fourth, significant differences between groups on current medication use were followed up in additional sensitivity analyses. Given the low number (*n* = 2) of antipsychotic (neuroleptic) medication, this variable was not included as a covariate in additional sensitivity analyses though higher rates in MDD compared to HCs. Given the importance of considering any medication use in endocrinological assessment (Granger, Hibel, Fortunato, & Kapelewski, 2009), future work in larger samples should consider these possible confounding effects. Fifth, comorbid mental disorders were unevenly distributed between groups and sexes with HCs not being free of lifetime mental disorders. Future studies should include participants with higher rates of comorbid mental disorders though sensitivity analyses showed no substantial influence of comorbid mental disorders on psychoneuroendocrine stress responses here. Also, heterogenous genetic and clinical characteristics (e.g., atypical, seasonal, melancholic subtypes of depression) may impact neurobiological findings (Lynch, Gunning, & Liston, 2020) and should be considered in future work. Lastly, though we have assessed major endocrine measures of the HPA, HPG axis, and neuropeptide system, future work should investigate whether findings also apply to other measures and earlier levels of these systems.

Keeping these limitations in mind, our findings point to a fundamentally changed psychoneuroendocrinological stress response in both girls and boys with MDD compared to healthy youth, which may influence their successful stress regulation and may thus be an important aspect to further investigate to improve treatment possibilities and success.

## Ethical considerations

Data were collected at the Goethe University Hospital Frankfurt am Main, Germany, with approval of the local ethical committee. Written informed consent was obtained from all participants and their legal guardians after study explanation.

**Key points**

- Exposure to psychosocial stress is one of the strongest risk factors for MDD in youth, but underlying neurobiological mechanisms are poorly understood.
- Different measures of psychoneuroendocrine stress responses (psychological, cortisol, testosterone, oxytocin) to the TSST in girls and boys with MDD compared to healthy adolescents were investigated.
- Both females and males with MDD showed a higher psychological, but blunted neuroendocrinological stress response of different neuroendocrine systems (HPA axis, HPG axis, neuropeptide system) compared to healthy youth.
- Findings point to a fundamentally changed psychoneuroendocrinological stress response in girls and boys with MDD, which may influence successful stress regulation in the affected adolescents and may thus be an important aspect to further investigate to improve treatment possibilities and success.

## Supporting information


**Appendix S1.** Additional method information – Procedures.
**Appendix S2.** Additional method information – Psychoneuroendocrine assessment.
**Appendix S3.** Additional method information – Repeated measures analyses of co‐variance (rmANCOVA).
**Table S1.** Sample description in participants with major depressive disorder (MDD) compared to healthy controls (HCs) for the oxytocin subsample.
**Table S2.** Psychoneuroendocrine levels in major depressive disorder (MDD) participants compared to healthy controls (HCs).
**Table S3.** Spearman‐correlations of psychoneuroendocrine stress response measures in participants with major depressive disorder (MDD) compared to healthy controls (HCs).
**Table S4.** Results of hierarchical linear models of psychoneuroendocrine stress response in participants with major depressive disorder (MDD) compared to healthy controls (HCs) considering additional potentially confounding effects.
**Figure S1.** Psychoneuroendocrine stress response to the Trier Social Stress Test in participants with major depressive disorder (MDD) compared to healthy controls (HCs) for log‐transformed levels of psychological stress (A), cortisol (B), testosterone (C), and oxytocin (D).
**Table S5.** Results of repeated measures analyses of co‐variance of psychoneuroendocrine stress responses in participants with major depressive disorder (MDD) compared to healthy controls (HCs).
**Table S6.** Results of exploratory repeated measures analyses of co‐variance of psychoneuroendocrine stress responses in participants with major depressive disorder (MDD) compared to healthy controls (HCs) considering effects of group, sex, and age.

## Data Availability

Data can be shared upon reasonable request.
