# Supplementary material for: Psychoneuroendocrine stress response in female and male youth with major depressive disorder
Source: J Child Psychol Psychiatry. 2025 Mar 23;66(10):1563–76. doi: 10.1111/jcpp.14168 (PMC12447675; doi:10.1111/jcpp.14168)
Supplement: Supplementary file 1 — Appendix S1. Additional method information – Procedures. Appendix S2. Additional method information – Psychoneuroendocrine assessment. Appendix S3. Additional method information – Repeated measures analyses of co‐variance (rmANCOVA). Table S1. Sample description in participants with major depressive disorder (MDD) compared to healthy controls (HCs) for the oxytocin subsample. Table S2. Psychoneuroendocrine levels in major depressive disorder (MDD) participants compared to healthy controls (HCs). Table S3. Spearman‐correlations of psychoneuroendocrine stress response measures in participants with major depressive disorder (MDD) compared to healthy controls (HCs). Table S4. Results of hierarchical linear models of psychoneuroendocrine stress response in participants with major depressive disorder (MDD) compared to healthy controls (HCs) considering additional potentially confounding effects. Figure S1. Psychoneuroendocrine stress response to the Trier Social Stress Test in participants with major depressive disorder (MDD) compared to healthy controls (HCs) for log‐transformed levels of psychological stress (A), cortisol (B), testosterone (C), and oxytocin (D). Table S5. Results of repeated measures analyses of co‐variance of psychoneuroendocrine stress responses in participants with major depressive disorder (MDD) compared to healthy controls (HCs). Table S6. Results of exploratory repeated measures analyses of co‐variance of psychoneuroendocrine stress responses in participants with major depressive disorder (MDD) compared to healthy controls (HCs) considering effects of group, sex, and age. [file JCPP-66-1563-s001.docx]

**Supplemental information for**

***“*****Psychoneuroendocrine Stress Response in Female and Male Youth with Major Depressive Disorder*”***

Anka Bernhard^1,2^, Nikola Fann^1^, Andreas G. Chiocchetti^1^, Katharina Ackermann^1,3^, Anne Martinelli^1,4^, & Christine M. Freitag^1^

^1^Department of Child and Adolescent Psychiatry, Psychosomatics and Psychotherapy, University Hospital Frankfurt am Main, Goethe University, Frankfurt am Main, Germany

^2^Department of Child and Adolescent Psychiatry and Psychotherapy, Faculty of Medicine, Technische Universität Dresden, German Center for Child and Adolescent Health (DZKJ), partner site Leipzig/Dresden, Dresden, Germany

^3^Faculty of Education, University Hamburg, Hamburg, Germany

^4^Fresenius University of Applied Sciences Frankfurt am Main, Frankfurt am Main, Germany

**Appendix S1.** Additional Method information - Procedures

Current and lifetime mental disorders according to DSM-IV-TR (American Psychiatric Association, 2000) criteria were assessed with the Kiddie-Schedule for Affective Disorders and Schizophrenia-Present and Lifetime version (K-SADS-PL; Kaufman et al., 1997), a semi-structured diagnostic interview conducted separately with adolescents and parents by trained staff. According to the K-SADS-PL instructions (Kaufman et al., 1997), the interviewer followed their best clinical judgment in assigning the summary ratings including all sources of information (e.g., adolescent, parent, school, clinic), particularly in case of discrepancies between sources. The most frequent disagreements occurred for items related to subjective phenomena (e.g., the adolescent described the presence or absence of certain symptoms, of which the parent wasn’t informed of or hadn’t observed, such as items of guilt, hopelessness, sleep disturbances, hallucinations, or suicidal ideation). If the disagreements were related to observable behaviors (e.g., truancy, fire setting, compulsive ritual), the interviewer queried sources about the discrepant information. Finally the interviewer was advised to use his or her best clinical judgment in assigning the summary ratings (Kaufman et al., 1997). If needed, the case would have also been discussed with experienced clinicians within the research team. The K-SADS-PL is a valid and reliable instrument with interrater agreement of 98% across all diagnoses and a test-retest reliability between 0.55 and 1.00, with highest values for Major Depressive Disorder (Cohen’s κ=.90 – 1.00) (Kaufman et al., 1997).

IQ was estimated from the matrix reasoning and vocabulary subtests of the Wechsler Intelligence Scale-IV (11-16 years: Wechsler, 2003); >16 years: Wechsler, 2008). Estimated IQ was defined as the arithmetic mean of the scored IQ-points of the two subscales.

Parental educational status was defined as the mean of the highest maternal and paternal self-reported school or occupational degree following International Standard Classification of Education (ISCED) criteria (Organisation for Economic Co-operation and Development, 1999). ISCED criteria for parental educational status are based on six categorical levels: 0=pre-primary level of education, 1=primary level of education, 2=lower secondary level of education, 3=upper secondary level of education, 4=post-secondary level of education, 5=first stage of tertiary education, 6=second stage of tertiary education.

Pubertal status was assessed using the Pubertal Development Scale (PDS; Petersen, Crockett, Richards, & Boxer, 1988), a self-report measure of pubertal growth (e.g. changes in body hair, voice or breast development) with four response options (not yet, barely started, definitely started, seems complete) resulting in a five-level categorical scale (0=pre-pubertal, all items not yet started; 1=early-pubertal, at least one item barely started; 2=mid-pubertal, at least one item definitely started; 3=late-pubertal, all items definitely started, 4=post-pubertal, all items seems complete). The internal consistency was *α*=0.77 (Petersen et al., 1988). Moderate to high associations with the clinician-rated Tanner stages support the reliability of the PDS (Koopman-Verhoeff et al., 2020).

Current body mass index (BMI; weight[kg]/height[m]²) of participants were calculated using measured weight (via weight scale) and height (via height measuring scale at the wall) at assessment time.

Current medication use (substance name, time of intake, dose) was assessed via self-report of participants about medication taken on the day of assessment and if reported, categorized as follows: antipsychotics (neuroleptics), stimulants (methylphenidate, amphetamine), non-stimulants (atomoxetine, guanfacine), selective serotonin reuptake inhibitor/antidepressants, tranquilizer (benzodiazepines), contraceptives, or other (e.g., vitamins, iron, pain killers, allergy/asthma medication). The category “Any medication” (yes, no) covers assignment to at least one of the above mentioned medication categories.

Current smoking of nicotine (cigarettes/day) was assessed via self-report of participants on the number of daily smoked cigarettes (“How many cigarettes do you smoke on an average day?”).

**Appendix S2.** Additional Method information - Psychoneuroendocrine assessment

Stress responsivity (see Figure 1) was assessed with the Trier Social Stress Test (TSST; Kirschbaum, Pirke, & Hellhammer, 1993), a widely-used, standardized method for inducing psychosocial stress in laboratory settings (Allen et al., 2014). Whenever possible when testing females, the date of the TSST procedure was arranged to occur during the second half of the menstrual cycle, in the luteal phase of menstruation, to increase comparability with males (Kajantie & Phillips, 2006). After a 60-minute relaxation period in a comfortable room (Room A) to minimize possible effects of previous food/drink intake, exercise, or stressful events, the baseline assessments were taken. Next, participants entered a sparsely-equipped, minimally distracting experimental room (Room B) and were given an introduction to the task. After a short preparation period (three minutes), they had to give a public speech (about their favorite movie including the storyline and their opinions of the characters and plot), followed by an age-adapted mental arithmetic task (serial subtraction of a fixed number from a given starting number, e.g., 13 from 1023, starting again from the beginning if they gave the wrong answer). Both tasks (each five minutes) were performed in front of two unfamiliar panel members who were trained and instructed to act in an emotionally neutral manner and provide no encouragement or positive feedback (Allen et al., 2014; Het et al., 2009). A video camera was also present and one of the panel members pretended to activate it at the start of the session. Post-stress assessments were completed in Room A, where participants were given positive feedback about their performance, informed that no recordings were made, and fully debriefed. Alcohol/substance use was not permitted on the assessment day nor drinking, eating, or smoking during the whole experiment. Standardized operating procedures for the TSST were used with adherence controlled by internal monitoring. To confirm stress induction, participants rated their feelings of stress (“Do you feel stressed?”) eight times from baseline to +55 minutes after stress termination using a Visual Analog Scale (range from 0=no, not at all to 10=yes, very much) (Hellhammer & Schubert, 2012). Saliva samples were collected using Salivettes (Sarstedt, Germany) at baseline and +1, +10, +25, +40 and +55 minutes after stress termination. Samples were stored at -20°C until analysis.

Levels of salivary cortisol (nmol/L) at baseline, +10, +25, +40 and +55 minutes, and testosterone (pg/ml) at baseline, +10 and +55 minutes were analyzed by daacro’s Saliva Lab Trier (Trier, Germany; https://daacro.de/en/saliva-lab-trier/) employing an enzyme immunoassay kit (Salimetrics, LLC, USA) formatted to minimize cross-reactivity for related steroids. The sensitivity limit was 0.19-82.77 nmol/L for cortisol and 1-600 pg/ml for testosterone. The corresponding inter- and intra-assay coefficients of variation were in the commonly accepted range <15% and <10%, respectively (Salimetrics, 2024).

After the cortisol and testosterone analyses, the baseline, +1 and +10 minutes Salivettes were transferred to RIAgnosis (Sinzing, Germany) for quantification of salivary oxytocin (pg/ml) by radioimmunoassay (RIA). For each sample, 300 µl of saliva was evaporated (Concentrator, Eppendorf, Germany) and 50µl of assay buffer was added, followed by 50µl rabbit antibody against oxytocin. After a 60 minutes preincubation interval, 10µl ^125^I-labeled tracer (PerkinElmer, Waltham, MA, USA) was added and samples incubated for three days at 4 ºC. Unbound radioactivity was precipitated by activated charcoal (Sigma-Aldrich, St Louis, MO, USA). Under these conditions, an average of 50% of total counts are bound with <5% non-specific binding. The detection limit of the RIA was in the 0.1-0.5 pg/sample range, depending on the age of the tracer, with typical displacements of 20–25% at 2 pg, 60–70% at 8 pg, and 90% at 32 pg of standard neuropeptide. Cross-reactivity with related neuropeptides were <0.7%. Intra- and inter-assay coefficients of variation were <10%. Serial dilutions of saliva samples containing high levels of endogenous oxytocin run strictly parallel to the standard curve indicating immuno-identity. Oxytocin was measured in a sub-sample of 143 participants (see Table S1), including 93 youth with MDD (58 female participants, 62.4%) and 50 HCs (28 female participants; 56.0%). Collection of samples at the +1 minute timepoint (optimal for oxytocin) commenced around one year into the study, after validation of salivary oxytocin stress responsiveness in youth (Bernhard et al., 2018). Different timepoints for cortisol, testosterone and oxytocin analyses were based on reported distinct reactivity patterns (Allen et al., 2014; Bernhard et al., 2018; de Jong et al., 2015).

**Appendix S3.** Additional Method information - Repeated measures analyses of co-variance (rmANCOVA)

While the hierarchical linear model approach provides numerous strengths (Dijkers, 2013), for easier comparison with previous work (Bernhard et al., 2021), classical repeated measures analyses of co-variance (rmANCOVA) were also performed for the main hypotheses. RmANCOVAs were performed using SPSS v29 (IBM Corp., Armonk, NY). Significance levels of all tests were set at *p*≤0.05 (two-tailed). Neuroendocrine measures were log-transformed to normalize their distribution. Dimensional covariates (age, BMI) were mean-centered (Delaney & Maxwell, 1981). Analyses of neuroendocrine measures were controlled for age, pubertal status, body mass index (BMI), and current smoking. Analyses of psychological stress were controlled for age and pubertal status only. Psychoneuroendocrine stress response was analyzed using four rmANCOVAs for the dependent variables psychological stress, cortisol, testosterone, and oxytocin with group (MDD vs. HCs) and sex (female vs. male) as between-subjects factors, and time as a within-subject factor. Greenhouse-Geisser corrections were applied as required (for clarity, uncorrected degrees of freedom are reported). Significant differences in psychoneuroendocrine stress responsivity were followed by post-hoc Bonferroni-corrected pair-wise comparisons. Effect sizes are described using partial eta squared *η*²_p_ with 0.01, 0.06, and 0.14 representing small, medium and large effects, respectively (Cohen, 1988). Incomplete data of seven participants on psychological stress (MDD: n=5, HCs: n=2) and of three participants on cortisol (MDD: n=2, HC: n=1) led to their exclusion in the respective rmANCOVA. For clarity, results of sample characteristics cover the full sample size, and did not change when excluding participants with missing data.

**Table S1.** Sample Description in Participants with Major Depressive Disorder (MDD) compared to Healthy Controls (HCs) for the Oxytocin subsample.

|  | Females (n=86) | | | | Males (n=57) | | | |  | |  | |  | | |
| --- | --- | --- | --- | --- | --- | --- | --- | --- | --- | --- | --- | --- | --- | --- | --- |
|  | MDD (n=58) | | HCs (n=28) | | MDD (n=35) | | HCs (n=22) | | Group | | Sex | | Group x sex | | |
|  | Mean (SD) or N (%) | | | | | | | | *p* | | *p* | | *p* | | |
| Age (years) | 15.26 (1.5) | | 15.11 (1.8) | | 15.37 (1.4) | | 14.91 (1.3) | | .25 | | .87 | | .56 | | |
| Estimated full-scale IQ | 106.21 (11.1) | | 106.43 (13.2) | | 106.64 (9.5) | | 103.41 (12.9) | | .46 | | .53 | | .40 | | |
| Parental educational status^a^ | 3.66 (1.0) | 4.20 (0.9) | | 3.38 (1.0) | | 3.48 (0.7) | | .06 | | <.01 | | .19 | |  |  |
| Body mass index | 23.4 (5.3) | | 20.8 (4.2) | | 25.1 (5.7) | | 21.21 (3.0) | | <.001 | | .24 | | .48 | | |
| Start time Trier Social Stress Test (hh:mm) | 15:16 (00:23) | | 15:12 (00:21) | | 15:03 (00:30) | | 15:11 (00:27) | | .65 | | .14 | | .21 | | |
| Pubertal status^a^ |  | |  | |  | |  | | .08 | | <.001 | |  | | |
| Mid-pubertal | 1 (1.7) | | 2 (7.1) | | 13 (37.1) | | 13 (59.1) | |  | |  | |  | | |
| Late-pubertal | 38 (65.5) | | 20 (71.4) | | 22 (62.9) | | 9 (40.9) | |  | |  | |  | | |
| Post-pubertal | 19 (32.8) | | 6 (21.4) | | 0 (0.0) | | 0 0.0) | |  | |  | |  | | |
| Current smoking | 22 (37.9) | | 1 (3.6) | | 7 (20.0) | | 2 (9.1) | | <.001 | | .12 | |  | | |
| Any medication | 46 (79.3) | | 6 (21.4) | | 27 (77.1) | | 3 (13.6) | | <.001 | | .35 | |  | | |
| Antipsychotics | 6 (10.3) | | 0 (0.0) | | 0 (0.0) | | 0 (0.0) | | .07 | | .04 | |  | | |
| Stimulants | 2 (3.4) | | 0 (0.0) | | 1 (2.9) | | 0 (0.0) | | .20 | | .82 | |  | | |
| Non-stimulants^b^ | 0 (0.0) | | 0 (0.0) | | 1 (2.9) | | 0 (0.0) | | .46 | | .22 | |  | | |
| SSRIs/antidepressants | 40 (69.0) | | 0 (0.0) | | 23 (65.7) | | 0 (0.0) | | <.001 | | .47 | |  | | |
| Tranquilizer | 1 (1.7) | | 0 (0.0) | | 0 (0.0) | | 0 (0.0) | | .46 | | .41 | |  | | |
| Contraceptives | 10 (18.2) | | 5 (20.8) | | 0 (0.0) | | 0 (0.0) | | .78 | |  | |  | | |
| Other | 16 (27.6) | | 3 (10.7) | | 10 (28.6) | | 3 (13.6) | | .03 | | .92 | |  | | |
| Comorbid mental disorders |  | |  | |  | |  | |  | |  | |  | | |
| Lifetime ADHD | 5 (8.6) | | 0 (0.0) | | 4 (11.4) | | 0 (0.0) | | .01 | | .67 | |  | | |
| Lifetime substance use disorder | 0 (0.0) | | 0 (0.0) | | 4 (11.4) | | 0 (0.0) | | .09 | | <.01 | |  | | |
| Lifetime anxiety disorder | 28 (48.3) | | 2 (7.1) | | 17 (48.6) | | 0 (0.0) | | <.001 | | .89 | |  | | |
| Lifetime PTSD | 9 (15.5) | | 0 (0.0) | | 2 (5.7) | | 1 (6.7) | | .05 | | .26 | |  | | |
| Lifetime eating disorder | 10 (17.2) | | 0 (0.0) | | 0 (0.0) | | 0 (0.0) | | .02 | | <.01 | |  | | |

*Note.* ^a^For definition of parental educational status and pubertal categories, see Appendix S1. ^b^Non-stimulants include atomoxetine medication. ’Other’ includes asthma medication, painkiller, or vitamin preparation. Participants may have been prescribed more than one medication category. ^c^Rates of lifetime anxiety disorder include lifetime panic disorder, separation anxiety disorder, avoidant disorder, simple phobia, social phobia, agoraphobia, and overanxious disorder/generalized anxiety disorder.

**Table S2**. Psychoneuroendocrine levels in Major Depressive Disorder (MDD) participants compared to healthy controls (HCs).

| Psychoneuroendocrine levels, Mean (SD) | Females (n=109) | | Males (n=66) | |
| --- | --- | --- | --- | --- |
|  | MDD (n=64) | HCs (n=45) | MDD (n=39) | HCs (n=27) |
| PSYCHOLOGICAL STRESS |  |  |  |  |
| VAS 1 (Baseline) | 3.05 (3.1) | 1.00 (1.6) | 2.83 (2.6) | 0.80 (1.3) |
| VAS 2 (+ 2 minutes) | 6.59 (3.2) | 2.75 (2.7) | 4.90 (2.6) | 1.60 (2.0) |
| VAS 3 (+ 7 minutes) | 7.24 (3.0) | 3.50 (2.9) | 5.16 (3.0) | 3.16 (3.0) |
| VAS 4 (+ 8 minutes) | 4.81 (3.2) | 1.22 (1.8) | 3.55 (3.5) | 0.89 (1.3) |
| VAS 5 (+ 18 minutes) | 3.35 (3.0) | 0.37 (0.7) | 2.34 (2.9) | 0.61 (1.2) |
| VAS 6 (+ 33 minutes) | 2.48 (2.7) | 0.34 (0.6) | 1.63 (1.9) | 0.42 (1.0) |
| VAS 7 (+ 48 minutes) | 1.88 (2.4) | 0.24 (0.5) | 1.65 (2.1) | 0.33 (0.6) |
| VAS 8 (+ 63 minutes) | 1.80 (2.5) | 0.35 (1.0) | 1.65 (1.8) | 0.21 (0.4) |
| AUC_I_ | 22.22 (12.5) | 7.10 (6.2) | 15.70 (12.3) | 6.06 (5.8) |
| NEUROENDOCRINE MEASURES |  |  |  |  |
| Cortisol (nmol/L) |  |  |  |  |
| Baseline | 3.66 (1.8) | 3.28 (2.1) | 3.31 (1.7) | 3.03 (1.5) |
| + 10 minutes | 5.32 (5.5) | 6.40 (4.3) | 4.48 (2.9) | 7.00 (6.5) |
| + 25 minutes | 4.57 (4.9) | 5.10 (3.7) | 4.96 (5.0) | 4.80 (3.9) |
| + 40 minutes | 3.71 (2.4) | 4.30 (2.2) | 4.23 (4.2) | 3.58 (2.4) |
| + 55 minutes | 3.53 (1.8) | 3.74 (1.8) | 3.33 (2.4) | 3.23 (1.6) |
| AUC_I_ | -1.11 (12.4) | 3.01 (12.6) | 0.44 (13.2) | 3.38 (10.4) |
| Testosterone (pg/ml) |  |  |  |  |
| Baseline | 45.55 (18.1) | 33.85 (13.4) | 91.4 (43.8) | 58.47 (29.2) |
| + 10 minutes | 49.74 (19.7) | 43.3 (19.7) | 99.8 (46.0) | 69.64 (37.5) |
| + 55 minutes | 48.47 (17.8) | 37.3 (16.2) | 98.4 (50.1) | 67.06 (34.1) |
| AUC_I_ | 5.65 (15.9) | 11.12 (13.7) | 11.93 (22.5) | 15.47 (23.8) |
| Oxytocin (pg/ml) |  |  |  |  |
| Baseline | 1.33 (0.3) | 1.55 (0.8) | 1.27 (0.3) | 1.17 (0.7) |
| + 1 minute | 1.46 (0.3) | 2.05 (1.5) | 1.40 (0.4) | 1.62 (0.9) |
| + 10 minutes | 1.49 (0.4) | 1.70 (1.0) | 1.36 (0.3) | 1.29 (0.7) |
| AUC_I_ | 0.22 (0.4) | 0.57 (1.65) | 0.17 (0.3) | 0.50 (0.8) |

*Note.* AUC_I_=Area under the curve with respect to increase, SD=Standard deviation.

**Table S3**. Spearman-correlations of psychoneuroendocrine stress response measures in participants with Major Depressive Disorder (MDD) compared to healthy controls (HCs).

| *AUC_I_* | Cortisol | Testosterone | Oxytocin |
| --- | --- | --- | --- |
| OVERALL |  |  |  |
| *All (N=165)* |  |  |  |
| Psychological stress | -.05 | -.10 | .03 |
| Cortisol | - | .44*** | -.04 |
| Testosterone |  | - | -.03 |
| *Females (N=102)* |  |  |  |
| Psychological stress | -.07 | -.09 | .06 |
| Cortisol | - | .50*** | -.11 |
| Testosterone |  | - | .03 |
| *Males (N=63)* |  |  |  |
| Psychological stress | .04 | -.06 | .05 |
| Cortisol | - | .31* | .07 |
| Testosterone |  | - | -.15 |
| MDD |  |  |  |
| *All (N=98)* |  |  |  |
| Psychological stress | .08 | -.09 | .08 |
| Cortisol | - | .42*** | -.07 |
| Testosterone |  | - | -.11 |
| *Females (N=61)* |  |  |  |
| Psychological stress | .19 | .08 | .15 |
| Cortisol | - | .52*** | -.17 |
| Testosterone |  | - | -.05 |
| *Males (N=37)* |  |  |  |
| Psychological stress | .05 | -.09 | -.06 |
| Cortisol | - | .23 | .14 |
| Testosterone |  | - | -.17 |
| HCs |  |  |  |
| *All (N=67)* |  |  |  |
| Psychological stress | .15 | .07 | .001 |
| Cortisol | - | .43*** | .002 |
| Testosterone |  | - | .10 |
| Females (N=41) |  |  |  |
| Psychological stress | .03 | .10 | -.17 |
| Cortisol | - | .49** | .02 |
| Testosterone |  | - | .17 |
| Males (N=26) |  |  |  |
| Psychological stress | .29 | -.02 | .37 |
| Cortisol | - | .36 | -.09 |
| Testosterone |  | - | -.15 |

*Note:* AUC_I_=Area under the curve with respect to increase. **p*<.05, ***p*<.01, ****p*<.001.

**Table S4**. Results of Hierarchical Linear Models of psychoneuroendocrine stress response in participants with Major Depressive Disorder (MDD) compared to healthy controls (HCs) considering additional potentially confounding effects.

|  |  | | | | Sensitivity Analyses | | | | |  |
| --- | --- | --- | --- | --- | --- | --- | --- | --- | --- | --- |
|  | *b* | *SE* | β | *p* | ADHD | ANX | PTSD | EAT | SSRI | OtherMed |
| PSYCH. STRESS |  |  |  |  |  |  |  |  |  |  |
| Sex | 0.17 | 0.12 | 0.08 | .14 | yes | yes | yes | yes | yes | yes |
| Group | 0.75 | 0.09 | 0.37 | **<.01** | yes | yes | yes | yes | yes | yes |
| Time (poly 1) | -8.28 | 1.01 | -0.22 | **<.01** | yes | yes | yes | yes | yes | yes |
| Time (poly 2) | 1.97 | 1.01 | 0.05 | **.05** | yes | yes | yes | yes | yes | yes |
| Group x time (poly 1) | -7.62 | 1.32 | -0.16 | **<.01** | yes | yes | yes | yes | yes | yes |
| Group x time (poly 2) | -1.15 | 1.32 | -0.02 | .38 | yes | yes | yes | yes | yes | yes |
| CORTISOL |  |  |  |  |  |  |  |  |  |  |
| Sex | 0.05 | 0.17 | 0.02 | .76 | yes | yes | yes | yes | yes | yes |
| Group | -0.04 | 0.14 | -0.02 | .79 | yes | yes | yes | yes | yes | yes |
| Time (poly 1) | 0.48 | 0.86 | 0.02 | .58 | yes | yes | yes | yes | yes | yes |
| Time (poly 2) | -7.95 | 0.86 | -0.27 | **<.01** | yes | yes | yes | yes | yes | yes |
| Group x time (poly 1) | -1.68 | 1.13 | -0.04 | .14 | yes | yes | yes | yes | yes | yes |
| Group x time (poly 2) | 3.84 | 1.13 | 0.10 | **<.01** | yes | yes | yes | yes | yes | yes |
| TESTOSTERONE |  |  |  |  |  |  |  |  |  |  |
| Sex | -1.15 | 0.15 | -0.56 | **<.01** | yes | yes | yes | yes | yes | yes |
| Group | 0.44 | 0.13 | 0.22 | **<.01** | yes | yes | yes | yes | yes | yes |
| Time (poly 1) | 1.12 | 0.49 | 0.05 | **.02** | yes | yes | yes | yes | yes | yes |
| Time (poly 2) | -3.13 | 0.49 | -0.14 | **<.01** | yes | yes | yes | yes | yes | yes |
| Group x time (poly 1) | -0.24 | 0.65 | -0.01 | .71 | yes | yes | yes | yes | yes | yes |
| Group x time (poly 2) | 1.75 | 0.64 | 0.06 | **.01** | yes | yes | yes | yes | yes | yes |
| OXYTOCIN |  |  |  |  |  |  |  |  |  |  |
| Sex | 0.30 | 0.20 | 0.14 | .14 | yes | yes | yes | yes | yes | yes |
| Group | -0.07 | 0.17 | -0.03 | .68 | yes | yes | yes | yes | yes | yes |
| Time (poly 1) | 3.20 | 1.33 | 0.14 | **.02** | yes | yes | yes | yes | yes | yes |
| Time (poly 2) | -5.88 | 1.33 | -0.26 | **<.01** | yes | yes | yes | yes | yes | yes |
| Group x time (poly 1) | -0.87 | 1.60 | -0.03 | .59 | yes | yes | yes | yes | yes | yes |
| Group x time (poly 2) | 5.01 | 1.60 | 0.18 | **<.01** | yes | yes | yes | yes | yes | yes |

*Note.* Hierachical linear models for the dependent variables psychological stress, cortisol, testosterone, and oxytocin, with the independent variable of group (MDD vs. HCs) interacting with a linear (polynomial 1) and quadratic (polynomial 2) time effect, controlled for major confounders as fixed effects (psychological stress: age, pubertal status; neuroendocrine measures: age, pubertal status, current smoking, body masss index). Columns of sensitivity analysis indicate whether effects hold the same significance level (yes: p<.05, no:>.05) as in the respective HLM without the additional covariate. ADHD=attention-deficit/hyperactivity disorder, ANX=anxiety disorders, EAT=eating disorders, PTSD=posttraumatic stress disorder, OtherMed=other medication includes usage of asthma medication, painkiller, or vitamin preparation, Psych.Stress=psychological stress, SSRI=selective serotonin reuptake inhibitor/ antidepressant medication. *η²_p_*=partial eta squared.

**
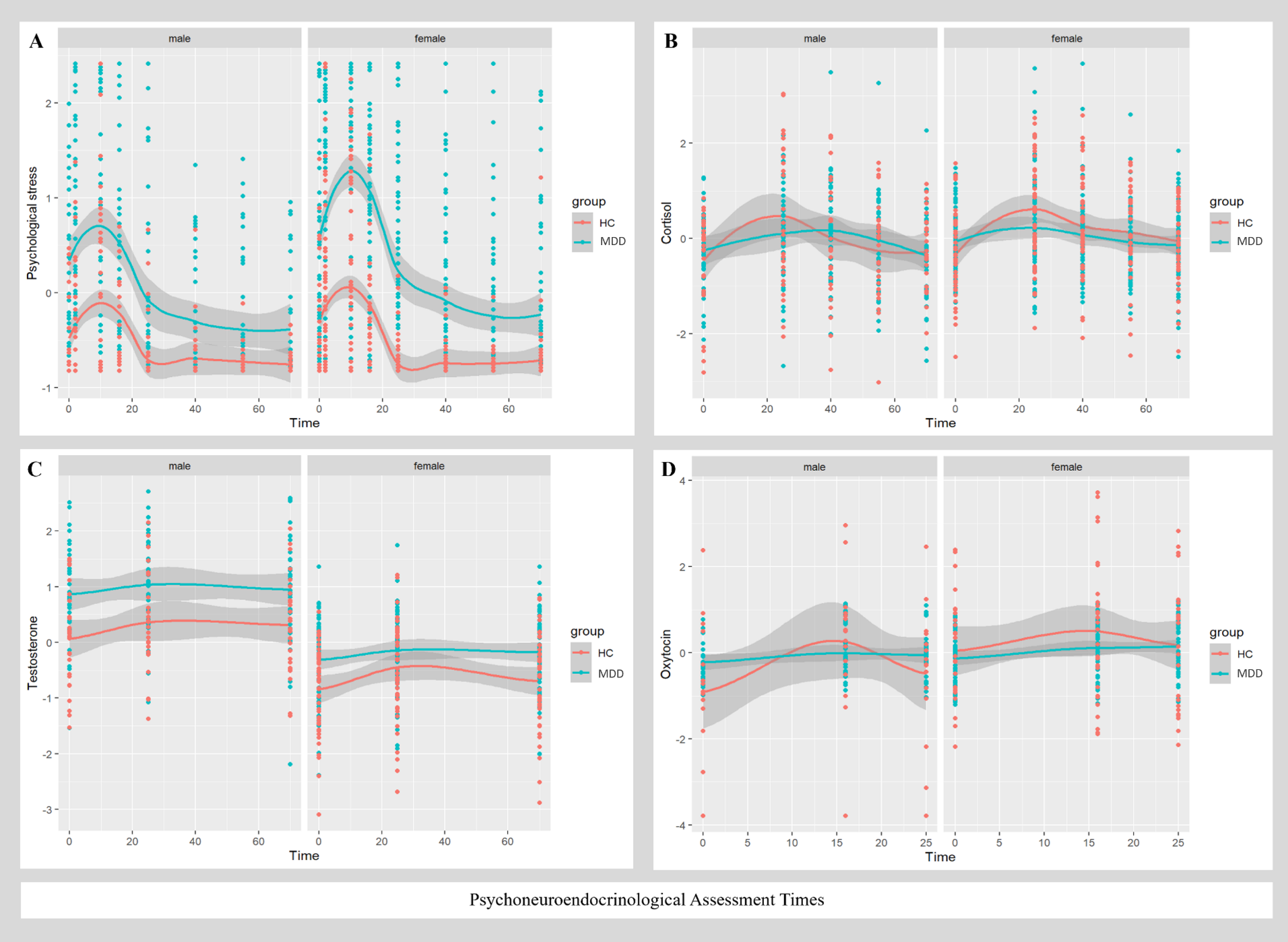
**

**Figure S1.** Psychoneuroendocrine stress response to the Trier Social Stress Test in participants with Major Depressive Disorder (MDD) compared to healthy controls (HCs) for log-transformed levels of psychological stress (A), cortisol (B), testosterone (C), and oxytocin (D). Data represent means + standard error measure.

**Table S5**. Results of repeated measures analyses of co-variance of psychoneuroendocrine stress responses in participants with Major Depressive Disorder (MDD) compared to healthy controls (HCs).

|  | Psychological stress | | | | Cortisol | | | | Testosterone | | | | | Oxytocin | | | |  |
| --- | --- | --- | --- | --- | --- | --- | --- | --- | --- | --- | --- | --- | --- | --- | --- | --- | --- | --- |
|  | *df* | *F* | *p* | *η²_p_* | *df* | *F* | *p* | *η²_p_* | *df* | *F* | *p* | *η²_p_* | *df* | | *F* | *p* | *η²_p_* | |
| Time | 7, 1134 | 112.33 | **<.001** | 0.41 | 4, 656 | 33.56 | **<.001** | 0.17 | 2, 328 | 20.45 | **<.001** | 0.11 | 2, 270 | | 14.56 | **<.001** | 0.10 | |
| Group | 1, 162 | 52.13 | **<.001** | 0.24 | 1, 164 | 0.09 | .77 | <0.01 | 1, 164 | 12.32 | **<.01** | 0.07 | 1, 135 | | <.01 | .98 | <0.01 | |
| Sex | 1, 162 | 1.46 | .23 | 0.01 | 1, 164 | 0.29 | .59 | <0.01 | 1, 164 | 52.04 | **<.001** | 0.24 | 1, 135 | | 3.64 | .06 | 0.03 | |
| Group x time | 7, 1134 | 8.05 | **<.001** | 0.05 | 4, 656 | 4.70 | **<.01** | 0.03 | 2, 328 | 1.65 | .20 | 0.01 | 2, 270 | | 3.71 | **.03** | 0.03 | |
| Sex x time | 7, 1134 | 1.63 | .16 | 0.01 | 4, 656 | 1.05 | .36 | 0.01 | 2, 328 | 1.39 | .25 | 0.01 | 2, 270 | | 0.90 | .41 | <0.01 | |
| Group x sex x time | 7, 1134 | 1.16 | .33 | 0.01 | 4, 656 | 1.50 | .22 | 0.01 | 2, 328 | 1.84 | .17 | 0.01 | 2, 270 | | 1.42 | .24 | 0.01 | |

*Note.* Repeated measures analyses of co-variance (rmANCOVA) with group (MDD vs. HCs) and sex (female vs. male) as between-subjects factors, and time as within-subject factor, respectively, controlled for major confounders (psychological stress: age, pubertal status; neuroendocrine measures: age, pubertal status, current smoking, body mass index). Where necessary, Greenhouse Geisser corrections were applied, but for clarity uncorrected degrees of freedom are reported here. *η²_p_*=partial eta squared.

**Table S6**. Results of exploratory repeated measures analyses of co-variance of psychoneuroendocrine stress responses in participants with Major Depressive Disorder (MDD) compared to healthy controls (HCs) considering effects of group, sex, and age.

|  | Psychological stress | | | | Cortisol | | | | Testosterone | | | | | Oxytocin | | | |  |
| --- | --- | --- | --- | --- | --- | --- | --- | --- | --- | --- | --- | --- | --- | --- | --- | --- | --- | --- |
|  | *df* | *F* | *p* | *η²_p_* | *df* | *F* | *p* | *η²_p_* | *df* | *F* | *p* | *η²_p_* | *df* | | *F* | *p* | *η²_p_* | |
| Time | 7, 980 | 52.90 | **<.001** | 0.27 | 4, 568 | 23.97 | **<.001** | 0.14 | 2, 248 | 18.49 | **<.001** | 0.12 | 2, 230 | | 25.20 | **<.001** | 0.18 | |
| Group | 1, 140 | 25.73 | **<.001** | 0.16 | 1, 142 | 0.12 | .73 | <0.01 | 1, 142 | 8.75 | **<.01** | 0.06 | 1, 115 | | 0.08 | .37 | <0.01 | |
| Sex | 1, 140 | 1.95 | .17 | 0.01 | 1, 142 | 1.26 | .26 | <0.01 | 1, 142 | 31.22 | **<.001** | 0.18 | 1, 115 | | 4.88 | **.03** | 0.04 | |
| Age | 6, 140 | 1.56 | .17 | 0.06 | 6, 142 | 1.80 | .10 | 0.07 | 6, 142 | 2.06 | .06 | 0.08 | 6, 115 | | 1.68 | .13 | 0.08 | |
| Sex x age | 6, 140 | 0.98 | .44 | .04 | 6, 142 | 0.46 | .84 | 0.02 | 6, 142 | 2.04 | .06 | 0.08 | 6, 115 | | 1.00 | .43 | 0.05 | |
| Group x sex | 1, 140 | 0.65 | .42 | <.01 | 1, 142 | 1.60 | .21 | 0.01 | 1, 142 | 0.04 | .84 | <0.01 | 1, 115 | | 1.48 | .23 | 0.01 | |
| Group x age | 6, 140 | 0.57 | .75 | 0.02 | 6, 142 | 1.30 | .26 | 0.05 | 6, 142 | 1.78 | .11 | 0.07 | 6, 115 | | 1.78 | .11 | 0.09 | |
| Group x sex x age | 12, 140 | 0.89 | .56 | 0.07 | 12, 142 | 0.94 | .51 | 0.07 | 12, 142 | 1.53 | .12 | 0.11 | 10, 115 | | 0.76 | .67 | 0.06 | |
| Group x time | 7, 980 | 6.17 | **<.001** | 0.04 | 4, 568 | 2.79 | .06 | 0.02 | 2, 248 | 3.19 | **.05** | 0.02 | 2, 230 | | 10.29 | **<.001** | 0.08 | |
| Sex x time | 7, 980 | 1.54 | .19 | 0.01 | 4, 568 | 1.71 | .18 | 0.01 | 2, 248 | <0.01 | .99 | <0.01 | 2, 230 | | 0.94 | .39 | <0.01 | |
| Age x time | 42, 980 | 1.36 | .12 | 0.06 | 24, 568 | 1.04 | .41 | 0.04 | 12, 248 | 0.84 | .61 | 0.03 | 12, 230 | | 3.04 | **<.001** | 0.14 | |
| Group x sex x time | 7, 980 | 0.54 | .71 | <0.01 | 4, 568 | 1.15 | .32 | <.01 | 2, 248 | 2.29 | .10 | 0.02 | 2, 230 | | 1.12 | .33 | 0.01 | |
| Group x age x time | 42, 980 | 0.65 | .90 | 0.03 | 24, 568 | 1.64 | .07 | .07 | 12, 248 | 1.21 | .28 | 0.05 | 12, 230 | | 3.10 | **<.001*** | 0.14 | |
| Group x sex x age x time | 84, 980 | 0.43 | 1.00 | 0.04 | 48, 568 | 1.17 | .26 | 0.09 | 24, 248 | 0.89 | .61 | 0.07 | 20, 230 | | 1.58 | .06 | 0.12 | |

*Note.* Repeated measures analyses of co-variance (rmANCOVA) with group (MDD vs. HCs), sex (female vs. male), and age as between-subjects factors, and time as within-subject factor, respectively (neuroendocrine measures controlled for current smoking, body mass index). Given the high correlation between pubertal status and age (*r*=.46, *p*<.001), pubertal status was not additionally included. RmANCOVA rather than hierarchical linear models were applied to reduce number of effects (time as a single factor rather than linear and quadratic variations) and increase statistical power. Where necessary, Greenhouse Geisser corrections were applied, but for clarity uncorrected degrees of freedom are reported here. *η²_p_*=partial eta squared.*Group differences of oxytocin stress response in older participants [*F*(1, 91)=4.80, *p*=.03, *η²p*=.05] stronger than in younger [*F*(1, 44)=0.95, *p*=.34, *η²p*=.02] participants (ANOVA of the area under the curve with respect to increase with group as independent variable controlled for current smoking and body mass index; median split of age: young 12-14 years, n=48; old 15-18 years, n=95).

**References**

Allen, A. P., Kennedy, P. J., Cryan, J. F., Dinan, T. G., & Clarke, G. (2014). Biological and psychological markers of stress in humans: Focus on the Trier Social Stress Test. *Neuroscience and Biobehavioral Reviews*, *38*, 94–124. https://doi.org/10.1016/j.neubiorev.2013.11.005

American Psychiatric Association. (2000). *Diagnostic and Statistical Manual of Mental Disorders, Fourth Edition (DSM-IV-TR)*. American Psychiatric Association.

Bernhard, A., Mayer, J. S., Fann, N., & Freitag, C. M. (2021). Cortisol response to acute psychosocial stress in ADHD compared to conduct disorder and major depressive disorder: A systematic review. *Neuroscience and Biobehavioral Reviews*, *127*, 899–916. https://doi.org/10.1016/j.neubiorev.2021.06.005

Bernhard, A., van der Merwe, C., Ackermann, K., Martinelli, A., Neumann, I. D., & Freitag, C. M. (2018). Adolescent oxytocin response to stress and its behavioral and endocrine correlates. *Hormones and Behavior*, *105*, 157–165. https://doi.org/10.1016/j.yhbeh.2018.08.010

Cohen, J. (1988). *Statistical Power Analysis for the Behavioral Sciences* (2nd ed.). Lawrence Earlbaum Associates.

de Jong, T. R., Menon, R., Bludau, A., Grund, T., Biermeier, V., Klampfl, S. M., Jurek, B., Bosch, O. J., Hellhammer, J., & Neumann, I. D. (2015). Salivary oxytocin concentrations in response to running, sexual self-stimulation, breastfeeding and the TSST: The Regensburg Oxytocin Challenge (ROC) study. *Psychoneuroendocrinology*, *62*, 381–388. https://doi.org/10.1016/j.psyneuen.2015.08.027

Delaney, H. D., & Maxwell, S. E. (1981). On using analysis of covariance in repeated measures designs. *Multivariate Behavioral Research*, *16*, 105–123.

Dijkers, M. P. (2013). Chasing Change: Repeated-Measures Analysis of Variance Is So Yesterday! *Archives of Physical Medicine and Rehabilitation*, *94*, 597–599. https://doi.org/10.1016/j.apmr.2012.11.018

Hellhammer, J., & Schubert, M. (2012). The physiological response to Trier Social Stress Test relates to subjective measures of stress during but not before or after the test. *Psychoneuroendocrinology*, *37*(1), 119–124. https://doi.org/10.1016/j.psyneuen.2011.05.012

Het, S., Rohleder, N., Schoofs, D., Kirschbaum, C., & Wolf, O. T. (2009). Neuroendocrine and psychometric evaluation of a placebo version of the ‘Trier Social Stress Test.’ *Psychoneuroendocrinology*, *34*, 1075–1086. https://doi.org/10.1016/j.psyneuen.2009.02.008

Kajantie, E., & Phillips, D. I. W. (2006). The effects of sex and hormonal status on the physiological response to acute psychosocial stress. *Psychoneuroendocrinology*, *31*(2), 151–178. https://doi.org/10.1016/j.psyneuen.2005.07.002

Kaufman, J., Birmaher, B., Brent, D., Rao, U., Flynn, C., Moreci, P., Williamson, D., & Ryan, N. D. (1997). Schedule for Affective Disorders and Schizophrenia for School-Age Children-Present and Lifetime Version (K-SADS-PL): initial reliability and validity data. *Journal of the American Academy of Child and Adolescent Psychiatry*, *36*(7), 980–988. https://doi.org/10.1097/00004583-199707000-00021

Kirschbaum, C., Pirke, K. M., & Hellhammer, D. H. (1993). The ’Trier Social Stress Test’-A Tool for Investigating Psychobiological Stress Responses in a Laboratory Setting. *Neuropsychobiology*, *28*, 76–81. https://doi.org/10.1159/000119004

Koopman-Verhoeff, M. E., Gredvig-Ardito, C., Barker, D. H., Saletin, J. M., & Carskadon, M. A. (2020). Classifying Pubertal Development Using Child and Parent Report: Comparing the Pubertal Development Scales to Tanner Staging. *Journal of Adolescent Health*, *66*(5), 597–602.

Organisation for Economic Co-operation and Development. (1999). *Classifying educational programmes: Manual for ISCED-97 implementation in OECD countries* (OECD (ed.)).

Pavlidi, P., Kokras, N., & Dalla, C. (2021). Antidepressants’ effects on testosterone and estrogens: What do we know? *European Journal of Pharmacology*, *899*(March), 173998. https://doi.org/10.1016/j.ejphar.2021.173998

Petersen, A. C., Crockett, L., Richards, M., & Boxer, A. (1988). A Self-Report Measure of Pubertal Status: Reliability, Validity, and Initial Norms. *Journal of Youth and Adolescence*, *17*(2), 117–133.

Salimetrics. (2024). Calculating inter-and intra-assay-coefficients of variability. *Https://Salimetrics.Com/Calculating-Inter-and-Intra-Assay-Coefficients-of-Variability/*.

Wechsler, D. (2003). *Wechsler Intelligence Scale for Children—Fourth Edition (WISC-IV)*. TX: Psychological Corporation.

Wechsler, D. (2008). *Wechsler Adult Intelligence Scale–Fourth Edition (WAIS–IV)*. TX: NCS Pearson 22.
